# Histone deacetylase-10 liberates spermidine to support polyamine homeostasis and tumor cell growth

**DOI:** 10.1016/j.jbc.2022.102407

**Published:** 2022-08-19

**Authors:** Tracy Murray Stewart, Jackson R. Foley, Cassandra E. Holbert, Glynis Klinke, Gernot Poschet, Raphael R. Steimbach, Aubry K. Miller, Robert A. Casero

**Affiliations:** 1 Sidney Kimmel Comprehensive Cancer Center, Johns Hopkins University School of Medicine, Baltimore, Maryland, USA; 2 Metabolomics Core Technology Platform, Center for Organismal Studies (COS), Heidelberg University, Heidelberg, Germany; 3 Biosciences Faculty, Heidelberg University, Heidelberg, Germany; 4 Cancer Drug Development, German Cancer Research Center (DKFZ), Heidelberg, Germany; 5 German Cancer Consortium (DKTK), Heidelberg, Germany

**Keywords:** polyamine, spermidine, N8-acetylspermidine, histone deacetylase-10 (HDAC10), tumor microenvironment (TME), difluoromethylornithine (DFMO), polyamine-blocking therapy, metabolism, microbiome, colorectal cancer, AG, aminoguanidine, DFMO, difluoromethylornithine, *N*^1^-AcSpd, *N*^1^-acetylspermidine, diAcSpd, *N*^1^,*N*^8^-diacetylspermidine, HDAC10, histone deacetylase-10, *N*^8^-AcSpd, *N*^8^-acetylated spermidine, Spd, spermidine, TME, tumor microenvironment

## Abstract

Cytosolic histone deacetylase-10 (HDAC10) specifically deacetylates the modified polyamine *N*^8^-acetylspermidine (*N*^8^-AcSpd). Although intracellular concentrations of *N*^8^-AcSpd are low, extracellular sources can be abundant, particularly in the colonic lumen. Extracellular polyamines, including those from the diet and microbiota, can support tumor growth both locally and at distant sites. However, the contribution of *N*^8^-AcSpd in this context is unknown. We hypothesized that HDAC10, by converting *N*^8^- AcSpd to spermidine, may provide a source of this growth-supporting polyamine in circumstances of reduced polyamine biosynthesis, such as in polyamine-targeting anticancer therapies. Inhibitors of polyamine biosynthesis, including α-difluoromethylornithine (DFMO), inhibit tumor growth, but compensatory uptake of extracellular polyamines has limited their clinical success. Combining DFMO with inhibitors of polyamine uptake have improved the antitumor response. However, acetylated polyamines may use different transport machinery than the parent molecules. Here, we use CRISPR/Cas9-mediated HDAC10-knockout cell lines and HDAC10-specific inhibitors to investigate the contribution of HDAC10 in maintaining tumor cell proliferation. We demonstrate inhibition of cell growth by DFMO-associated polyamine depletion is successfully rescued by exogenous *N*^8^-AcSpd (at physiological concentrations), which is converted to spermidine and spermine, only in cell lines with HDAC10 activity. Furthermore, we show loss of HDAC10 prevents both restoration of polyamine levels and growth rescue, implicating HDAC10 in supporting polyamine-associated tumor growth. These data suggest the utility of HDAC10-specific inhibitors as an antitumor strategy that may have value in improving the response to polyamine-blocking therapies. Additionally, the cell-based assay developed in this study provides an inexpensive, high-throughput method of screening potentially selective HDAC10 inhibitors.

A class IIb cytosolic HDAC, histone deacetylase-10 (HDAC10), functions as a polyamine deacetylase, with high specificity for acetylated polyamines, particularly *N*^8^-acetylated spermidine (*N*^8^-AcSpd), over histones and other lysine-containing peptides (1). Acetylation of polyamines generally serves to reduce their positive charge, facilitating membrane transport (2). Nuclear *N*^8^-acetylation of spermidine (Spd) is believed to facilitate its transport to the cytoplasm, where HDAC10 mediates its deacetylation back to spermidine (1, 3, 4, 5). HDAC10 has been shown to have roles in autophagy, differentiation, DNA repair, exocytosis, and chemoresistance (6, 7, 8, 9), as well as epigenetic regulation (10). However, none of these roles have been clearly linked to its function as a polyamine deacetylase.

Polyamine homeostasis is dysregulated in nearly all cancer types, and targeting polyamine metabolism for cancer prevention and treatment has been a longstanding therapeutic strategy that has achieved some success (11). Difluoromethylornithine (DFMO) is an irreversible inhibitor of ornithine decarboxylase, the first rate-limiting step in polyamine biosynthesis (Fig. 1). Approved for the treatment of African trypanosomiasis, DFMO has a proven safety record and is a promising chemopreventive agent (12, 13). However, compensatory mechanisms exist through which tumor cells respond to reduced intracellular polyamine biosynthesis by increasing import of polyamines from the extracellular environment. Extracellular polyamines, including those in the tumor microenvironment (TME), derive from sources including diet, metabolic products of the microbiota, and sloughed or damaged cells, particularly in the intestinal lumen (14). *N*^8^-AcSpd is rarely detected to accumulate intracellularly but has been detected in extracellular fluids in association with certain pathological states (15, 16, 17, 18). Additionally, it is a metabolite of certain types of bacteria (19). Although generally regarded as an excretory metabolite, we considered that *N*^8^-AcSpd, via HDAC10-mediated deacetylation to spermidine, may serve as a source of polyamines to proliferating cells with increased polyamine requirements, particularly in the context of polyamine-limiting anticancer therapies.

**Figure 1:**
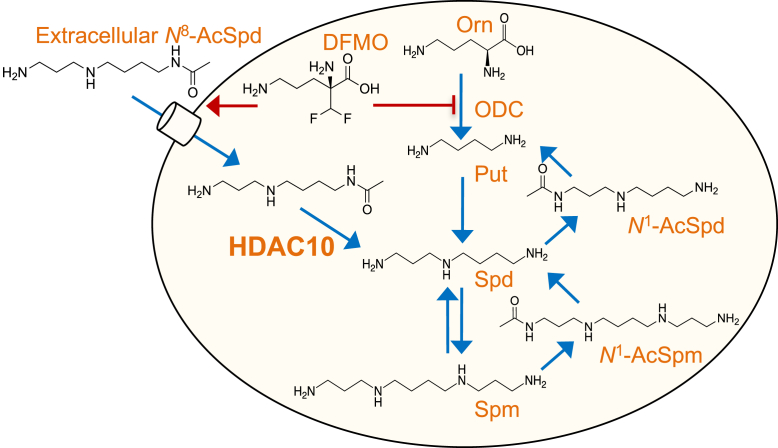
**Schematic of the role of HDAC10 in supporting mammalian polyamine metabolism.** Diflouromethylornithine (DFMO) inhibits ornithine decarboxylase (ODC), limiting polyamine biosynthesis and stimulating polyamine uptake from the tumor microenvironment in support of proliferation. Cytoplasmic HDAC10 converts imported *N*^*8*^*-*AcSpd into spermidine (Spd), which can be converted to spermine (Spm) or catabolized to putrescine (Put) via acetylation on its *N1* position followed by oxidation, thereby re-establishing polyamine homeostasis. HDAC10, histone deacetylase-10; *N*^8^-AcSpd, *N*^8^-acetylated spermidine.

## Results

### Knockout of *HDAC10* increases intracellular *N*^*8*^*-*AcSpd concentrations

**Figure 2:**
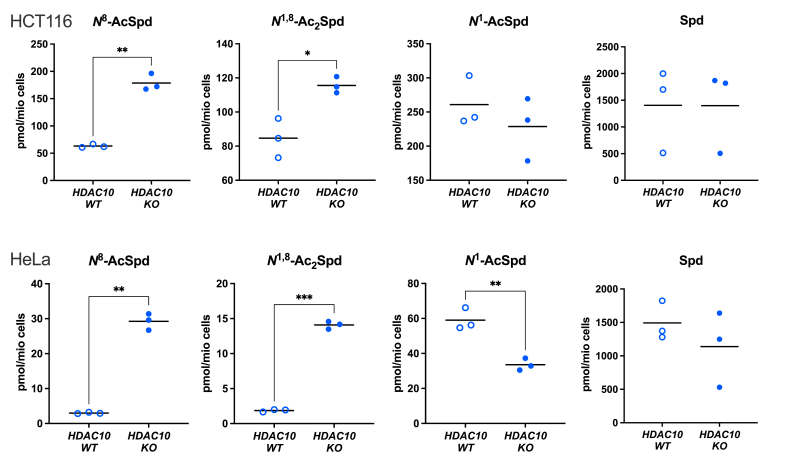
***HDAC10* knockout alters intracellular levels of acetylated Spd derivatives.** Targeted metabolomics data of wildtype (WT) and CRISPR/Cas9-generated HDAC10 knockout (KO) HCT116 (top) and HeLa S3 (bottom) cells. Statistically significant differences in metabolite concentrations between KO vs. WT are indicated as ∗ *p* ≤ 0.05, ∗∗*p* ≤ 0.01, and ∗∗∗*p* ≤ 0.001. HDAC10, histone deacetylase-10.

CRISPR/Cas9-mediated knockout of HDAC10 in HCT116 cells was evidenced by Western blotting (Fig. S1) followed by Sanger sequencing to confirm biallelic generation of indels. HeLa cell clones lacking HDAC10 were previously reported (20). Mass spectrometry analyses verified significantly increased levels of *N*^8^-AcSpd in both *HDAC10*-KO cell lines (Fig. 2). HCT116 cells had greater concentrations of this derivative in both WT and KO lines than did HeLa cells. Both cell lines also had significant increases in *N*^1^,*N*^8^-diacetylspermidine (diAcSpd), and the extent of this increase tended to correlate with reduction of *N*^1^-acetylspermidine (*N*^1^-AcSpd). No significant differences were detected in spermidine concentrations with *HDAC10* knockout in either cell line. These results are consistent with those observed in a neuroblastoma cell line with *HDAC10*-targeting siRNA (20) and confirm an association of *N*^8^-AcSpd with HDAC10 in cell lines derived from multiple tissues.

### Extracellular *N*^*8*^-AcSpd can support growth under polyamine-limiting conditions

Treatment of tumor cells with DFMO generally leads to a cytostatic response resulting from the depletion of intracellular polyamines. The import of extracellular polyamines can rescue this growth inhibition; however, whether *N*^8^-AcSpd maintains this ability was unknown.

**Figure 3:**
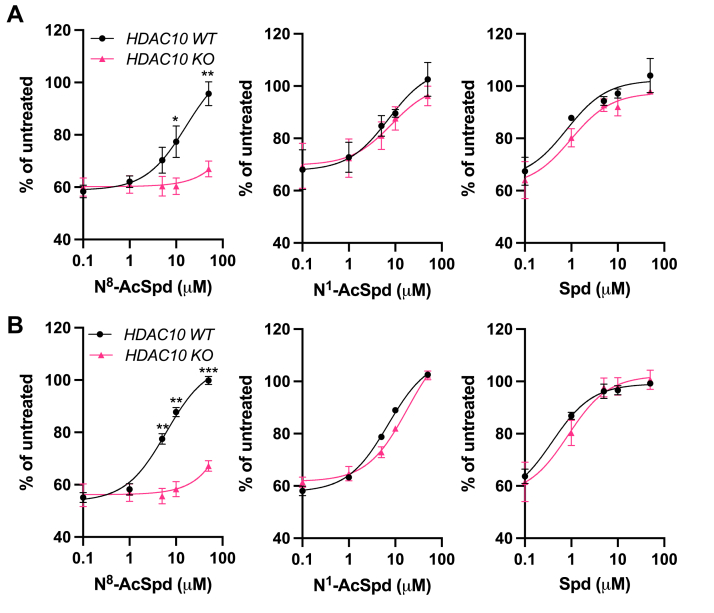
**HDAC10 is required for rescue of DFMO-mediated cell growth inhibition by *N***^**8**^**-AcSpd.** Inhibition of polyamine biosynthesis with DFMO causes growth inhibition that can be rescued by exogenous polyamines. *HDAC10* WT and KO HCT116 (*A*) and HeLa (*B*) cells were cotreated for 96 h with 5 mM DFMO and increasing concentrations of the indicated polyamine, with 1 mM aminoguanidine to prevent extracellular polyamine oxidation by bovine serum amine oxidase. Growth rescue was measured as a function of CellTiter-Blue fluorescence and is presented relative to cells not treated with DFMO. Loss of *HDAC10* prevented rescue by *N*^8^-AcSpd but had no effect on rescue by *N*^1^-AcSpd or unmodified Spd, consistent with the reported substrate specificity of purified protein. Statistically significant differences in rescue between WT and KO cells are as indicated by ∗*p* ≤ 0.05, ∗∗*p* ≤ 0.01, and ∗∗∗*p* ≤ 0.001. DFMO, diflouromethylornithine; HDAC10, histone deacetylase-10; KO, knockout; *N*^8^-AcSpd, *N*^8^-acetylated spermidine; WT, wildtype.

Cotreatment of *HDAC10*^*WT*^ HCT116 cells with DFMO and *N*^8^-AcSpd, in the presence of aminoguanidine to inhibit extracellular amine oxidation (21), demonstrated that this metabolite could rescue growth, while no rescue occurred in *HDAC10*^*KO*^ cells (Fig. 3*A*), suggesting a role for HDAC10 in allowing *N*^8^-AcSpd to re-enter the polyamine metabolic pathway in support of proliferation (Fig. 1). HDAC10 status had no effect on rescue by either *N*^1^-AcSpd or unmodified Spd, consistent with its high specificity for the *N*8 modification. These results are consistent with those obtained using *HDAC10*^*WT*^ and *HDAC10*^*KO*^ HeLa cells (Fig. 3*B*). Although providing monoacetylated spermidine rescued growth in WT cells regardless of which end of spermidine was modified, *N*^1^,*N*^8^-diAcSpd failed to support proliferation in the presence of DFMO (Fig. S2).

Studies with recombinant HDAC10 also indicated deacetylase activity for *N*-acetylputrescine (*N*-AcPut) (1). However, substituting *N*-AcPut for *N*^8^-AcSpd in our growth assays provided rescue regardless of the presence of HDAC10 (Fig. S2), consistent with alternative pathways for *N*-AcPut metabolism beyond HDAC10. As with Spd, unmodified putrescine (Put) also rescued DFMO-induced growth inhibition in both WT and KO cell lines.

### HDAC10 is essential in re-establishing polyamine homeostasis via *N*^8^-AcSpd

**Figure 4:**
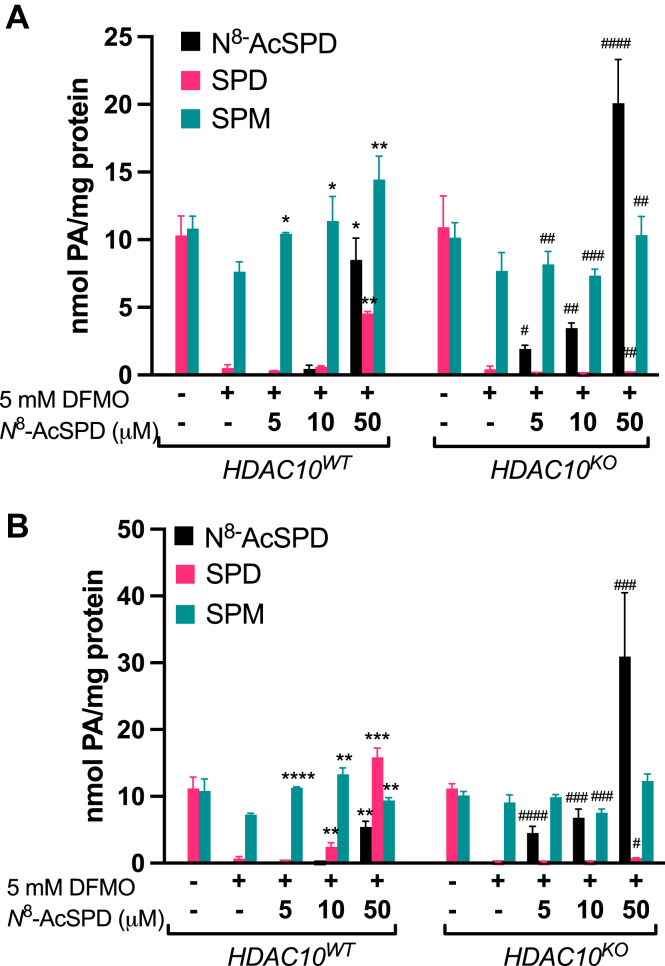
**Extracellular *N***^**8**^**-AcSpd functions as a source of polyamines for mammalian cells via HDAC10-mediated deacetylation.** In *HDAC10*^*WT*^ HCT116 (*A*) and HeLa (*B*) cells with DFMO-associated polyamine depletion, extracellular *N*^8^-AcSpd is rapidly converted to replenish SPD and SPM pools and rescue proliferation. In *HDAC10*^*KO*^ cells, *N*^8^-AcSpd is accumulated without conversion to SPD, coincident with the continuation of growth inhibition. ∗ indicates statistical significance relative to DFMO-treated *HDAC10*^*WT*^ cells; *#* indicates statistical significance of KO cells relative to the same treatment condition in WT cells. ∗ or ^#^*p* ≤ 0.05, ∗∗ or ^##^*p* ≤ 0.01, ∗∗∗ or ^###^*p* ≤ 0.001, ∗∗∗∗ or ^####^*p* ≤ 0.0001. DFMO, diflouromethylornithine; HDAC10, histone deacetylase-10; KO, knockout; *N*^8^-AcSpd, *N*^8^-acetylated spermidine.

The growth rescue provided by *N*^8^-AcSpd in the presence of HDAC10 suggested its deacetylation into Spd, which can then enter the polyamine metabolic pathway to re-establish polyamine homeostasis (Fig. 1). We therefore analyzed intracellular polyamine concentrations following the combination treatments in *HDAC10* WT and KO cell lines (Fig. 4). Treatment with DFMO similarly depleted Spd in all cell lines. In cell lines with WT HDAC10, treatment with *N*^8^-AcSpd led to a dose-dependent restoration of both Spd and spermine (Spm) levels, with very little accumulation of the acetylated metabolite below the highest dose level (50 μM). In contrast, *N*^8^-AcSpd markedly accumulated in *HDAC10*^*KO*^ cells at all concentrations tested, and no significant conversion to Spd was evident, supporting HDAC10 as the sole enzyme responsible for the deacetylation of *N*^8^-AcSpd. These data indicate that HDAC10 is an essential component in the metabolism of *N*^8^-AcSpd into Spd, allowing its subsequent interconversion within the polyamine metabolic pathway to restore growth-promoting polyamine homeostasis.

### Inhibition of HDAC10 activity suppresses colon cancer cell growth rescue by *N*^8^-AcSpd

**Figure 5:**
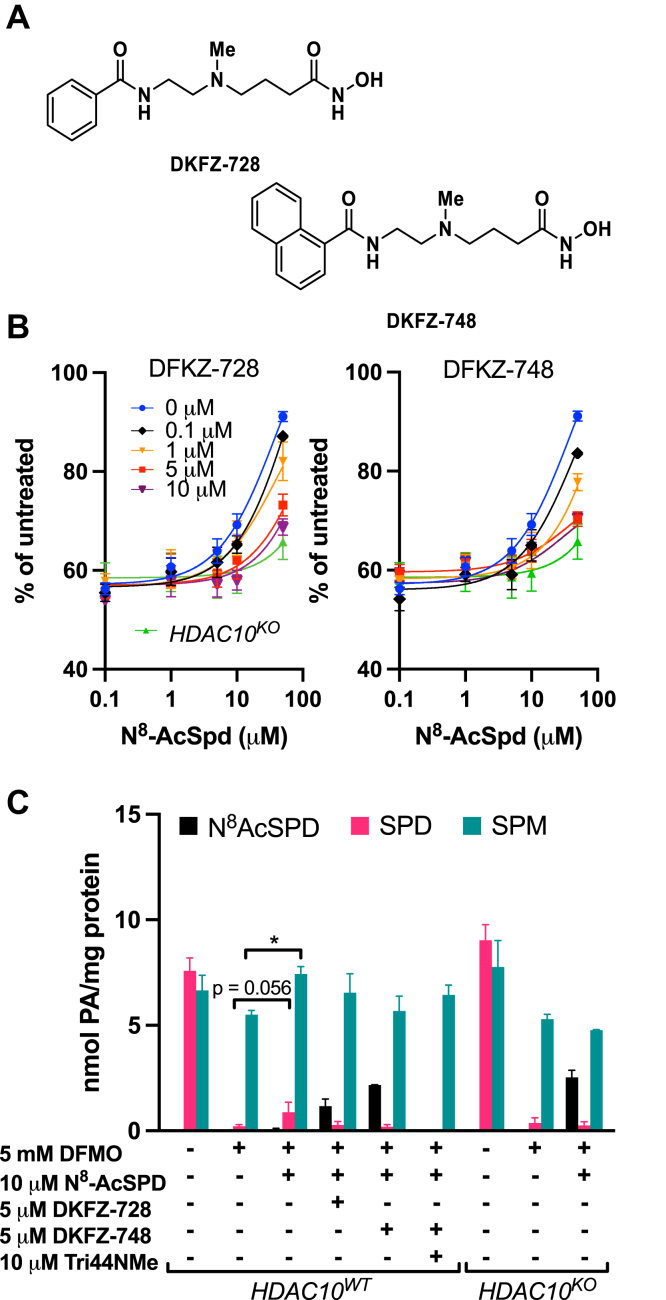
**HDAC10-selective inhibitors prevent rescue of DFMO-mediated cytostasis and restoration of polyamine homeostasis by *N***^**8**^**-AcSpd.***A*, structures of DKFZ-728 and DKFZ-748 HDAC10 inhibitors. *B*, HDAC10 inhibitors dose-dependently blocked rescue of cell growth by *N*^8^-AcSpd. HCT116 WT cells were pretreated 24 h with concentrations of HDAC10 inhibitors DKFZ-728 or DKFZ-748 ranging from 0.1 to 10 μM, followed by 96-h cotreatments with 5 mM DFMO, HDAC10 inhibitor, 1 mM AG, and increasing concentrations of *N*^8^-AcSpd. Percent growth relative to untreated cells was determined using CellTiter Blue–based fluorescence. *C*, pharmacological HDAC inhibition was as effective as genetic knockout in preventing entry of *N*^8^-AcSpd into the polyamine metabolic pathway. Treatment with the polyamine transport inhibitor Trimer44NMe prevented rescue by *N*^8^-AcSpd. HCT116 cells were treated as for growth assays and polyamines were determined by HPLC. ∗ indicates significantly increased intracellular polyamine concentration (Spd or Spm; *p* ≤ 0.05) compared to that of cells treated with DFMO alone. DFMO, diflouromethylornithine; HDAC10, histone deacetylase-10; *N*^8^-AcSpd, *N*^8^-acetylated spermidine.

*N*^8^-AcSpd is a metabolite present in feces and the intestinal lumen (19). We therefore extended our recent studies using HDAC10-selective inhibitors (20) to determine if inhibiting deacetylase activity was sufficient to prevent polyamine restoration and growth rescue by *N*^8^-AcSpd in HCT116 colon cancer cells. DKFZ-728 and DKFZ-748 (Fig. 5*A*) are newly described aza-SAHA derivatives with high biochemical and cellular specificity for HDAC10 over other HDAC isozymes. Incorporating these HDAC10 inhibitors into our growth rescue assays indicated dose-dependent reductions in the ability of *N*^8^-AcSpd to allow proliferation in HCT116 cells (Fig. 5*B*). DKFZ-748 was more effective at preventing rescue than DKFZ-728, consistent with previous biochemical studies indicating greater inhibition of HDAC10 activity by DKFZ-748 (20). Indeed, at doses of 5 and 10 μM DKFZ-748, the number of cells proliferating in the presence of *N*^8^-AcSpd did not significantly differ from those with genetic knockout of *HDAC10*, suggesting that inhibition of HDAC10 enzymatic activity phenocopies genetic knockout. The structurally related, negative control compound DKFZ-828, which does not have an inhibitory effect on HDAC10 activity, similarly had no inhibitory effect on the rescue of cell growth by *N*^8^-AcSpd (Fig. S3).

### Pharmacological HDAC10 inhibition prevents restoration of polyamine homeostasis by *N*^8^-AcSpd

To confirm that the inability of *N*^8^-AcSpd to rescue DFMO-mediated growth suppression in the presence of HDAC10 inhibitors is due to continued polyamine depletion, we analyzed intracellular polyamine concentrations following treatment in HCT116 cells (Fig. 5*C*). Both DKFZ-728 and DKFZ-748 effectively prevented the conversion of *N*^8^-AcSpd into Spd, as seen by the intracellular accumulation of the acetylated metabolite, with little increase in Spd or Spm levels. Although DKFZ-748 was again slightly more effective than DKFZ-728, the results with both inhibitors were comparable to those of *HDAC10*^*KO*^ cells, further confirming that pharmacologically inhibiting HDAC10 activity reproduces the phenotype observed with its genetic elimination in colorectal cancer cells and that HDAC10 is the sole enzyme responsible for deacetylating *N*^8^-AcSpd.

Cells were also treated with Trimer44NMe, a polyamine transport inhibitor (PTI) often used in conjunction with DFMO in polyamine-blocking strategies (22, 23, 24, 25), to determine its ability to block the import of acetylated spermidine. In both HCT116 and HeLa cells, adding the PTI prevented the uptake of *N*^8^-AcSpd, as evidenced by the lack of its conversion to spermidine as well as the absence of *N*^8^-AcSpd accumulation in the presence of the HDAC10 inhibitor (Figs. 5*C* and S4). Furthermore, no rescue of DFMO-mediated growth arrest by *N*^8^-AcSpd was evident in the presence of the PTI, which tended to exacerbate the growth inhibitory effects. These results demonstrate that the polyamine-blocking strategy of combining DFMO and Trimer44NMe sufficiently blocks uptake of *N*^8^-AcSpd, in addition to the unmodified polyamines.

## Discussion

Under normal conditions, *N*^8^-AcSpd exists at low levels in cells and tissues and is believed to be rapidly converted to spermidine by a cytosolic polyamine deacetylase that is now known to be HDAC10 (1, 4, 26). *N*^8^-AcSpd is, however, abundant in certain TMEs. Generally regarded as excretory metabolites, acetylated polyamines such as *N*^8^-AcSpd are normal components of urine and feces (19, 27, 28, 29). As they have been found to be elevated in association with certain pathologies, they have also been proposed as potential biomarkers, particularly for cancer (30, 31). Enrichment of intracellular *N*^8^-AcSpd has been reported in tumor tissue of colorectal cancer patients, while it was undetectable in adjacent normal tissue (32). Similarly, urinary concentrations of *N*^8^-AcSpd in colorectal cancer patients was significantly greater than in healthy volunteers but did not significantly differ from that of patients with benign gastrointestinal disease, most of which were chronic inflammatory conditions associated with malignant potential (32). Increased intracellular levels of *N*^8^-AcSpd have also been measured in colonic intestinal epithelial cells (IECs) taken from inflammatory bowel disease (IBD) patients at the most severely inflamed mucosal areas (33), suggesting increased uptake. Breakdown of the mucosal barrier during IBD pathogenesis allows increased exposure of IECs to microbiota and metabolites, including endogenous polyamines released by necrotic or damaged cells. As colonic epithelial cells must proliferate and migrate to repair the epithelial barrier, their requirement for polyamines is increased, likely stimulating both biosynthesis and uptake (34). A recent study directly demonstrated that microbiota-derived polyamines can be incorporated into host colonocytes to accelerate epithelial renewal (35).

Our studies indicate the important potential that extracellular acetylated polyamines, specifically *N*^8^-AcSpd, may serve as a source of polyamines for rapidly proliferating cells with increased requirements for polyamines – a nearly ubiquitous characteristic of tumor cells. Age-related changes in the composition of the microbiota are especially apparent in elderly populations and are associated with chronic low levels of inflammation. A comparison of fecal metabolites in elderly patients found elevated levels of *N*^8^-AcSpd in a patient population with an “elderly” type gut microbiota *versus* those with an age-mismatched, “adult” type microbiota generally associated with a younger population (11.6 nmol/g feces v. 7.4 nmol/g feces, respectively) (19). Notably, these authors also showed that treatment of HCT116 colon cancer cells with *N*^8^-AcSpd induced expression of pro-inflammatory cytokines and prevented oxaliplatin-mediated cytotoxicity. It is not currently known if a role exists for HDAC10 in these gene expression changes.

*N*^8^-AcSpd has also been shown to increase with age in the rat kidney, alongside an increase in spermidine acetyltransferase activities (36). As *de novo* polyamine biosynthesis decreases with age in many tissues (37), it has been suggested that maintaining acetylated spermidine pools, including *N*^8^-AcSpd, might compensate by increasing polyamine interconversion (36). In fact, HDAC10 is highly expressed in the kidney, as well as the spleen and liver (38). Similarly, analyses of plasma metabolites in the long-lived naked mole rat revealed elevated *N*^8^-AcSpd levels, compared to mice of similar developmental stage, in the absence of the age-related decline in spermidine observed in other mammals (39).

The role of HDAC10 in supporting malignancy appears to be context dependent and may relate to the availability of *N*^8^-AcSpd in the TME. Expression levels of HDAC10 in cancer patients are seen to correlate both favorably and unfavorably with patient prognosis, depending on tumor origin (40). Interestingly, Kaplan-Meier curves available at the Human Protein Atlas (proteinatlas.org) (40) demonstrate that cancers in which high HDAC10 expression is associated with poor prognosis include those that are associated with TMEs enriched in excretory products, including renal cancer and colon adenocarcinoma. A recent analysis of TCGA data reported HDAC10 copy number gains as a risk factor for colorectal and pancreatic cancers (41). A study of colon cancer patients in China reported constitutively elevated expression of HDAC10 protein in tumor tissues compared to paired adjacent tissue in the majority of patients (9), with the greatest difference observed in cytoplasmic HDAC10. Interestingly, while the expression level of HDAC10 in the tumor samples failed to correlate with clinicopathological factors, HDAC10 expression in the adjacent normal tissue positively correlated with the presence of lymph node and/or distant metastases. Further, while high cytoplasmic expression of HDAC10 in these tumors was predictive of good prognosis, high cytoplasmic expression in normal adjacent tissues predicted poor prognosis, and high nuclear HDAC10 expression in adjacent normal tissues correlated with improved survival (9). Similarly, in lung cancer cells, HDAC10 was observed to localize in the cytoplasm, while its detection was predominantly nuclear in adjacent normal cells (42). These data suggest the importance of subcellular HDAC10 localization, which hints at the catalytic mechanism of action based on substrate availability, as *N*^*8*^-AcSpd deacetylation occurs in the cytoplasm. Conversely, nuclear localization of HDAC10 may be associated with its potential histone deacetylase activity (1, 10).

Purified HDAC10 protein has been shown to deacetylate *N*^1^,*N*^8^-diAcSpd (1), and our results showing increased levels of this metabolite in *HDAC10*-KO cells provide evidence of this reaction *in situ*. The increased abundance of *N*^1^,*N*^8^-diAcSpd in cells lacking HDAC10 appeared to correlate with decreases in the pools of *N*^1^-AcSpd, suggesting that HDAC10-mediated deacetylation of diAcSpd may be an alternative source of *N*^1^-AcSpd, in addition to the *N*^1^-acetylation of spermidine by SSAT. However, diAcSpd failed to rescue DFMO-mediated growth inhibition even in cells with WT HDAC10, suggesting two possibilities: 1) *N*^1^,*N*^8^-diAcSpd is not metabolized into a growth-supporting polyamine moiety; or 2) it is not transported into the cell, as suggested by competition studies with tritiated spermidine (43). As our studies and others have indicated that *N*^1^-AcSpd can rescue DFMO-mediated growth arrest (44), the latter is most likely. Arylamine *N-*acetyltransferase (NAT2) was recently reported to acetylate *N*^1^-AcSpd at the *N*8 position in colon cancer cells, producing *N*^*1*^*,N*^*8*^-diAcSpd (45). Enzymatic assays indicated that NAT2 can also acetylate *N*^8^-AcSpd to produce the diacetylated molecule. Thus, the increase in diAcSpd might be secondary to the increased abundance of *N*^8^-AcSpd in the absence of HDAC10. Regardless, extracellular *N*^1^,*N*^8^-diAcSpd in the TME appears to be a metabolic end product incapable of supporting tumor cell growth.

A main limitation of anticancer strategies targeting polyamine biosynthesis, particularly the use of DFMO, is the compensatory increase in polyamine uptake from the extracellular environment in order to maintain homeostasis (11). Blocking this restoration of homeostasis is the goal of polyamine-blocking therapies, which incorporate the simultaneous inhibition of biosynthesis and transport (23, 25, 46, 47, 48). Several studies have indicated this to be a promising strategy, with effects on tumor cells as well as tumor-associated immune cells (14, 49). However, mammalian polyamine transport is complex, and its mechanisms have not been fully elucidated. It is possible that *N*^8^-AcSpd, with its reduced charge, may not be a substrate for the same polyamine transporter that imports spermidine and spermine in mammalian cells. The commonly used PTI Trimer44NMe effectively blocked growth rescue and restoration of polyamine homeostasis by *N*^8^-AcSpd in our studies, suggesting that it also blocks transport of acetylated spermidine molecules. However, as the complexities of the polyamine transport system are still being elucidated, we emphasize that this finding should not be broadened to include all PTIs or all cell types as certain cell types appear to express multiple polyamine transporters with different substrate affinities (25, 47). On this basis, we speculate that specific inhibition of HDAC10 may improve the efficacy of polyamine-blocking therapy by blocking an alternative source of spermidine in certain TMEs.

In conclusion, our studies indicate an essential role for HDAC10 in the conversion of *N*^8^-AcSpd to spermidine. To our knowledge, this is the first demonstration that *N*^8^-AcSpd can serve to re-establish polyamine homeostasis in colorectal cancer cells exposed to polyamine-limiting conditions. Our data indicate HDAC10-mediated deacetylation of exogenous polyamines as an alternative source of tumor-supporting polyamines capable of bypassing polyamine-limiting therapies, such as DFMO, emphasizing the need to consider this pathway in the design of polyamine-blocking strategies and suggesting that HDAC10-specific inhibitors may be a beneficial addition to such strategies in certain contexts. Additionally, our cell-based growth rescue assay provides an inexpensive method for future screening of potential HDAC10 inhibitors.

## Experimental procedures

### Cell culture and reagents

HCT116 (CCL-247) and HeLa S3 (CCL-2.2) cells were obtained from ATCC. HCT116 cells were grown in McCoy’s 5A medium with 10% fetal bovine serum (Gemini Bio Products) and penicillin/streptomycin solution (Corning). HeLa S3 cells were grown in MEM-alpha (Corning) with 10% fetal bovine serum, sodium pyruvate, and penicillin/streptomycin solution (Corning). Cell lines were maintained in a humidified atmosphere at 37°C, 5% CO_2_ and were routinely screened for mycoplasma infection using MycoAlert (Lonza).

Aminoguanidine (AG; 1 mM) was used in all studies including the addition of exogenous polyamines to limit extracellular polyamine oxidation by bovine serum amine oxidase (21). DFMO was provided by Dr Patrick Woster at the Medical University of South Carolina. AG, *N*^8^-AcSpd, *N*^1^-AcSpd, *N*-AcPut, Put, Spd, and Spm were purchased from Sigma Chemical Co. *N*^1^,*N*^8^-diAcSpd was purchased from Cayman Chemical Co. The polyamine transport inhibitor Trimer44NMe was provided by Dr Otto Phanstiel (University of Central Florida).

### CRISPR-Cas9-mediated HDAC10 knockout

Oligonucleotides targeting the *HDAC10* gene were synthesized, annealed, and ligated into the single guide RNA scaffold of the LentiCRISPRv2-Blast vector (AddGene) via the BsmBI sites, according to methods previously described (50). Three different oligo pairs were chosen for construct synthesis targeting the following sequences in exon 2 (sgRNA one and 2) or 11 (sgRNA3) of *HDAC10*:

sgRNA1 AGTGCGAGATCGAGCGTCCTG

sgRNA2 GGATCGCCTGCGGCAGCGC

sgRNA3 GCATGACAGTACAGACGCTGC

Successful construct generation was verified by Sanger sequencing of the sgRNA region. Transfections of HCT116 and HeLa cells were performed using Lipofectamine 3000 (Invitrogen) followed by single-cell isolation through limiting dilution arrays and selection in growth media containing 10 μg/ml blasticidin. Lysates of individual CRISPR-Cas9-edited clones were prepared in 4% SDS for HDAC10 knockout screening by Western Blot. Proteins (30 μg/lane) were separated on 4 to 12% Bolt Bis-Tris polyacrylamide gels in MES running buffer (Invitrogen) and transferred onto Immun-blot PVDF membranes (Bio-Rad). Primary antibodies to HDAC10 (1:1000; Sigma #H3413) and PCNA (1:4000; Calbiochem #NA03) were incubated overnight at 4°C with rocking; IR-dye conjugated secondary antibodies (1:10,000; LI-COR #926–32211 and 1:20,000; ThermoFisher Scientific #A-21057) allowed detection using a LI-COR Odyssey imager with ImageStudioLite analysis software (LI-COR). Biallelic knockout through indel generation was confirmed in each cell line following PCR amplification of genomic DNA (extracted using Zymo Quick gDNA Micropreps) with primers flanking the guide RNA target of the *HDAC10* gene. PCR products were TOPO-TA cloned into the pCR4 vector for sequencing according to the manufacturer’s protocol (Invitrogen). All primers were synthesized by IDT, and Sanger sequencing was performed by the Genetic Resources Core Facility of the Johns Hopkins University School of Medicine. Individual clones with successful *HDAC10* knockout via each of the three targeted sequences were analyzed for variations in polyamine concentrations by HPLC. As no significant difference was detected, cell lines with biallelic indels in *HDAC10* exon 2 (targeted by sgRNA1 above) were chosen for subsequent experiments.

### Targeted metabolomics of acetylated spermidine

A targeted metabolomics approach was used to quantify acetylated polyamines in the HDAC10-knockout cells via UPLC-MS/MS. Each cell line was plated in triplicate 15-cm culture dishes at a density of 4 million cells/dish. After approximately 48 h, cells were collected, centrifuged, and resuspended in PBS with an aliquot taken for cell counts using trypan blue exclusion and a hemacytometer. Two cell pellets containing at least 10 million and 5 million cells each were produced from each culture dish and quick-frozen in a dry ice/ethanol bath for storage at -80°C. Extraction and quantification of acetylated spermidine was performed by UPLC-MS/MS as previously described (20).

### Cell proliferation studies

Cells were seeded in triplicate at 1000 cells/well of 96-well plates and allowed to attached overnight. Medium was aspirated and replaced with 100 μl of fresh medium containing 1 mM AG, 5 mM DFMO, and increasing concentrations of *N*^8^-AcSpd, Spd, *N*^1^-AcSpd, *N*-AcPut, Put, or *N*^1^,*N*^8^-diAcSpd. After incubation for 96 h, 20 μl of CellTiter-Blue reagent (Promega) was added and cells were incubated for an additional 2 h. Fluorescence was measured (560_Ex_/590_Em_) on a SpectraMax M5 (Molecular Devices). In experiments using HDAC10 inhibitors, cells were pretreated 24 h with the inhibitor (added approximately 4 h post seeding), after which the medium was replaced with that containing inhibitor, DFMO, and *N*^8^-AcSpd for an additional 96-h incubation.

### HPLC analysis of polyamine concentrations

Cells were seeded at 0.6 x 10^5^ cells per T-25 flask and allowed to attached overnight. Media was aspirated and replaced with that containing 1 mM AG, 5 mM DFMO, and *N*^8^-AcSpd at final concentrations of 5, 10, or 50 μM, followed by incubation for 96 h. Cell lysates were collected and acid-precipitated, and supernatants were used for precolumn derivatization of polyamines with Dansyl chloride (Sigma) in the presence of diaminoheptane as an internal standard. For experiments including HDAC10 inhibitors, cells were seeded as above and allowed to adhere to the flask approximately 4 h, at which time the inhibitor was added. After a 24-h pretreatment, the media was aspirated and refreshed with that containing inhibitor, DFMO, AG, and *N*^8^-AcSpd, for an additional 96-h incubation. Polyamines were quantified by HPLC as previously described (51) and are presented relative to total cellular protein in the lysate, as determined based on the method of Bradford (Bio-Rad Protein Assay), with interpolation on a bovine serum albumin standard curve.

### Statistical analyses

All statistically significant results were determined using GraphPad Prism (v. 9.3.1). Results are indicative of at least two independent biological replicates with at least duplicate determinations of each. Significant differences in polyamine concentrations between HDAC10 KO vs. WT were determined by an unpaired *t* test with Welch’s correction. Significant differences in cell growth rescue were calculated between WT and KO cells at each concentration by two-way ANOVA with the Sidak test for multiple comparisons. Differences in individual polyamine concentrations following rescue were determined by two-way ANOVA with Fisher’s LSD test. Designations for significance in all cases are the following: ∗ *p*-value ≤ 0.05, ∗∗ *p* ≤ 0.01, ∗∗∗ *p* ≤ 0.001.

## Data availability

All data are contained within the manuscript.

## Supporting information

This article contains supporting information.

## Conflict of interest

Work in the Casero and Stewart laboratory is supported, in part, by a research contract from Panbela Therapeutics, Inc.

## Supporting information

Murray Stewart SI
