# Supplementary material for: Histone deacetylase-10 liberates spermidine to support polyamine homeostasis and tumor cell growth
Source: J Biol Chem. 2022 Aug 19;298(10):102407. doi: 10.1016/j.jbc.2022.102407 (PMC9486564; doi:10.1016/j.jbc.2022.102407)
Supplement: Murray Stewart SI [file mmc1.pdf]

Histone deacetylase 10 liberates spermidine to support polyamine homeostasis and tumor cell growth

Tracy Murray Stewart[1], Jackson R. Foley[1], Cassandra E. Holbert[1], Glynis Klinke[2], Gernot Poschet[2], Raphael R. Steimbach[3,4], Aubry K. Miller[4,5], Robert A. Casero, Jr.[1]*

Supporting Information

Page	
S-2	Figure S1 Western blot of HDAC10 knockout
S-3	Figure S2 Growth rescue by N^1,N^8-diAcSpd, N-AcPut, Put, Spd
S-4	Figure S3 Negative control compound and effect on rescue
S-5	Figure S4 Trimer44NMe blocks N8-AcSpd uptake in HeLa cells

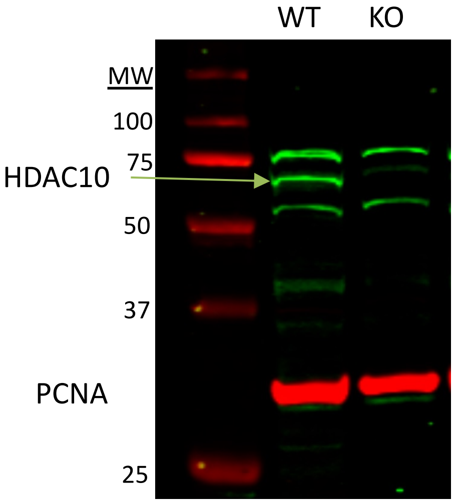

Figure S1. Western blot showing loss of HDAC10 protein (~72 kDa) following CRISPR/Cas9-mediated targeting in HCT116 cells. Proliferating cell nuclear antigen (PCNA) is used as a loading control.

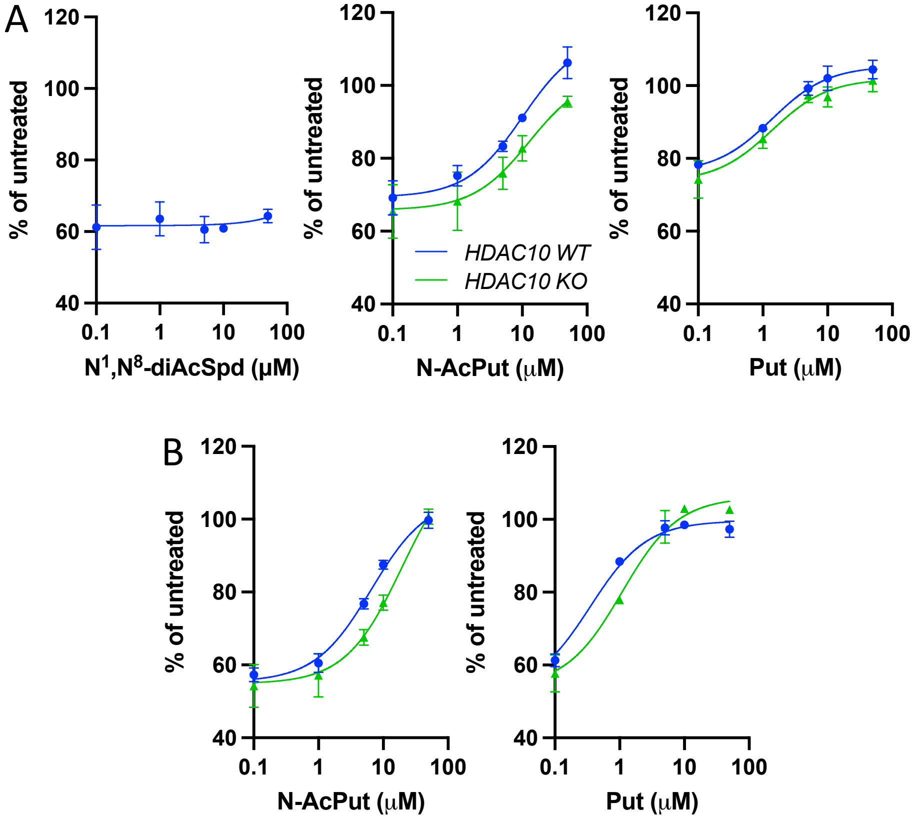

Figure S2. HDAC10 knockout has no effect on growth rescue by *N*[1],*N*[8]-diAcSpd, acetylated putrescine (*N*-AcPut) or putrescine (Put). HCT116 (A) or HeLa (B) cells were cotreated with 5 mM DFMO, 1 mM aminoguanidine, and increasing polyamine concentrations for 96 h. Proliferation was measured as a function of CellTiter Blue fluorescence.

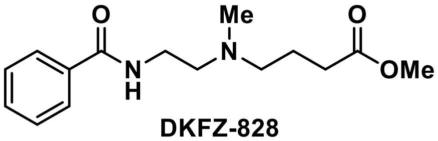

DKFZ-828

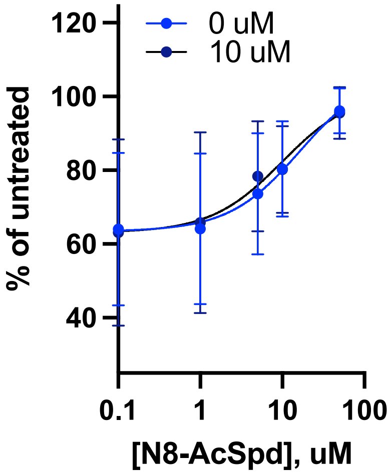

Figure S3. Inactive control compound DKFZ-828, which is structurally related to HDAC10 inhibitors DKFZ-728 and 748 but lacking HDAC10 inhibitory activity failed to block growth rescue by N^8-AcSpd in HCT116 cells.

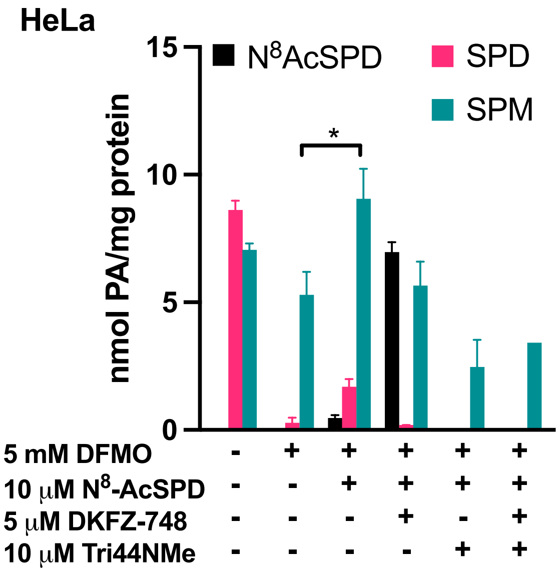

Figure S4. The polyamine transport inhibitor Trimer44NMe blocks uptake of N8-AcSpd in Hela cells.
